# Virus-like particles with FLAG-tagged envelope protein as a tetravalent dengue vaccine candidate

**DOI:** 10.1038/s41598-021-97038-4

**Published:** 2021-09-02

**Authors:** Toshifumi Imagawa, Masahiko Ito, Mami Matsuda, Kenji Nakashima, Yuhei Tokunaga, Isao Ohta, Tian-Cheng Li, Ryosuke Suzuki, Tetsuro Suzuki

**Affiliations:** 1 Department of Virology and Parasitology, Hamamatsu University School of Medicine, Hamamatsu, 431-3192 Japan; 2 Department of Virology II, National Institute for Infectious Disease, Musashi-murayama, Tokyo 208-0011 Japan; 3 Advanced Research Facilities and Services, Preeminent Medical Photonics Education and Research Center, Hamamatsu University School of Medicine, Hamamatsu, 431-3192 Japan

**Keywords:** Vaccines, Virology

## Abstract

The global incidence of dengue, which is caused by dengue virus (DENV) infection, has grown dramatically in recent decades and secondary infection with heterologous serotype of the virus may cause severe symptoms. Efficacious dengue vaccines should be able to provide long-lasting immunity against all four DENV serotypes simultaneously. In this study, we constructed a novel vaccine platform based on tetravalent dengue virus-like particles (DENV-LPs) in which envelope (E) protein carried a FLAG tag sequence at the position located not only in the exterior loop on the protruding domain but outside of dimerization interface of the protein. We demonstrated an effective strategy to produce the DENV-LPs by transient transfection with expression plasmids for pre-membrane and E proteins of DENV-1 to DENV-4 in mammalian cells and to concentrate and purify them with one-step affinity chromatography. Characteristic features of VLPs such as particle size, shape and density were comparable to flavivirus-like particles reported. The neutralizing activity against all four DENV serotypes was successfully induced by immunization with the purified tetravalent VLPs in mice. Simple, one-step purification systems for VLP vaccine platforms using epitope-tagging strategy should be advantageous for vaccine development not only for dengue but for emerging pandemics in the future.

## Introduction

Dengue is a mosquito-borne viral disease caused by dengue virus (DENV) and is widespread in particular throughout the tropics. The incidence of dengue has grown in recent decades and there are estimated 100–400 million infections each year^1^. Vector mosquitos have expanded their habitat in context of global warming and urbanization^2^. The clinical features of dengue vary from a non-specific febrile illness to at times fatal serious conditions such as dengue haemorrhagic fever and dengue shock syndrome^3^. Severe dengue is a leading cause of serious illness in some Asian and Latin American countries. There is no specific treatment for dengue/severe dengue to date.

DENV belongs to the Flavivirus genus of the Flaviviridae family, and there are four distinct serotypes; DENV-1 to DENV-4^4^. Antibody-dependent enhancement (ADE) of DENV infection, in which suboptimal neutralizing or non-neutralizing cross-reactive antibodies bind to virus and facilitate Fcγ receptor mediated enhanced entry into host cells, followed by increasing the cellular viral load, is thought to be the highest risk factor for dengue haemorrhagic fever and dengue shock syndrome^4,5^.

The DENV particle contains a positive-sense single-stranded RNA genome in their capsid which is surrounded by a lipid bilayer envelope that anchors two viral glycoproteins pre-membrane (prM) and envelope (E). The E protein, which is involved in receptor binding, is folded into three structurally distinct domains (Domains I–III) according to X-ray crystallographic studies^6^. Domain I is located in the middle of the protein, which is flanked by domains II and III. Domain II plays a major role in dimerization of E protein and harbors the hydrophobic fusion loop. Domain III can be folded as an immunoglobulin-like structure that is involved in host cell binding. This domain contains serotype-specific epitopes and makes up a relatively stable conformation, suggesting importance as a virus-specific antigen.

The E protein is the major target for antibody-mediated neutralization and thus the focus of vaccine design^7^. Due to the possible phenomenon of ADE in which infection with heterogenous serotype induces severe dengue pathogenesis, the challenge for development of dengue vaccine has been to generate a tetravalent vaccine that induces protective immunity against DENV-1, DENV-2, DENV-3, and DENV-4, simultaneously^8^.

CYD-TDV, a tetravalent live attenuated with a yellow fever backbone is the first dengue vaccine to be licensed in late 2015^9^. It was shown that the rate of severe dengue infection after immunization with CYD-TDV potentially increased in dengue antibody negative children compared to dengue antibody positive children^10^. The first licensed vaccine led to controversy, thus no dengue vaccine is in widespread use to date.

Virus-like particle (VLP) has been developed as one of new candidates of dengue vaccine in several studies. Since VLPs have structural and physicochemical features comparable to infectious particles, it is expected that immunogenicity is comparable to mature virion and it is not needed to consider occurrence of the vaccine derived strain. In fact, immunization with Dengue VLP successfully induced anti-DENV antibody^11,12^ and cytotoxic T cell response^11,13^, whereas it appears that the ADE of DENV infection was not induced by the VLP vaccination at least in mouse models^14,15^. Dengue VLPs have been produced by several protein expression systems using such as mammalian cells^11,12^, yeast Pichia pastoris^14^, Escherichia coli^16^, also silkworm transfected with recombinant baculovirus genome^17^. VLPs produced by recombinant expression systems have been purified through stepwise processes such as clarification, precipitation, ultrafiltration and chromatography.

In this study, we identified an optimal insertion site for FLAG tag sequence in DENV E protein for extracellularly production of DENV-LPs derived from four serotypes and established a rapid one-step purification system for DENV-LPs using the FLAG affinity purification technology. Further we demonstrated that tetravalent vaccination with purified DENV-LPs elicited neutralizing activities against all four serotypes of DENV simultaneously in mice.

## Results

### Expression of DENV VLPs carrying FLAG-tag in E protein

**Figure 1:**
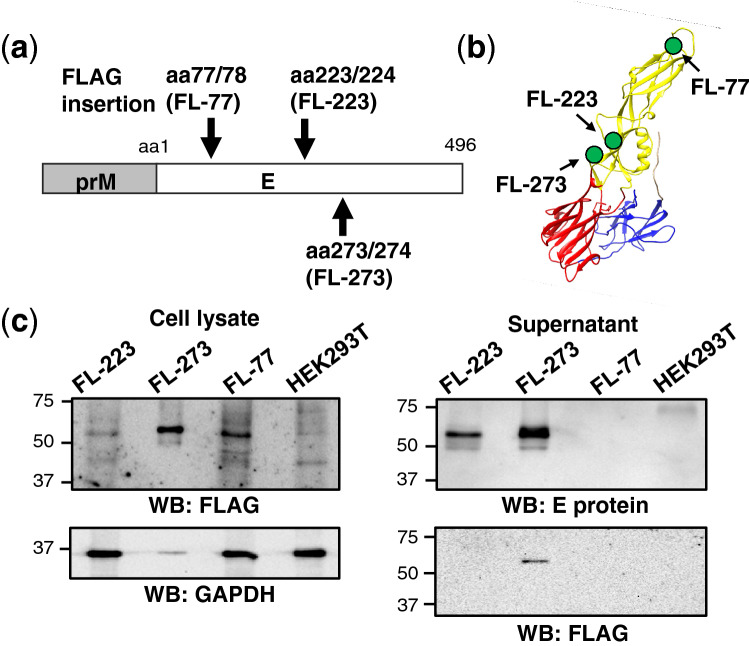
FLAG tag insertion at three positions of DENV E protein domain II in DENV-1 VLP expression plasmid. (**a**) Amino acid positions of FLAG tag insertion in E protein of DENV-1 are shown. (**b**) The 3D structure of DENV-1 E protein (aa 2–404 region) was constructed and three positions of FLAG tag insertion were shown. Circle symbols indicate FLAG tag insertion positions at aa 77/78, 223/224 and 273/274. Three domains in E protein were distinguished in red (domain I), yellow (domain II) and blue (domain III). (**c**) Expression of DENV-1 E protein with FLAG tag insertion from prME plasmids FL-223, -273 and -77 in HEK293T cells (left) and their culture supernatants (right) were compared by western blotting (WB). Monoclonal antibodies against FLAG tag (FLAG), flavi group antigen (E protein), and GAPDH were used as 1st antibodies. Non-transfected HEK293T cell lysate and culture supernatant samples were added as a negative control. Because of difference in expression efficiency among prME plasmids tested, amounts of samples applied for WB which produced from FL-273 were one-hundredth (cell lysate) and one-tenth (supernatant) compared to those from FL-223 and FL-77. The amounts of cell lysates applied were 3 mg for FL-223, FL-77 and HEK293T and 0.03 mg for FL-273. Uncropped images of western blotting pictures are included in supplementary Fig. 1.

To identify an optimal insertion site for foreign epitope in DENV E protein, we designed three prME expression plasmids carrying the FLAG epitope tag sequence either between amino acid (aa) residues 77 and 78 (FL-77), 223 and 224 (FL-223), or 273 and 274 (FL-273), located within the loop regions in DENV-1 E protein (Fig. 1a, b), based on the three-dimensional structure and the former study on Japanese encephalitis virus (JEV) sub-viral particles containing FLAG tag or hepatitis C virus-neutralizing epitope^18^. Since these three insertion sites located within domain II of the E protein are predicted to be located in the exterior loop on the protruding domain of the protein and spatially distant from domain III which is regarded as an important epitope for neutralizing activity^19^, an impact by the foreign epitope insertion on the viral antigenic response appears limited.

**Figure 2:**
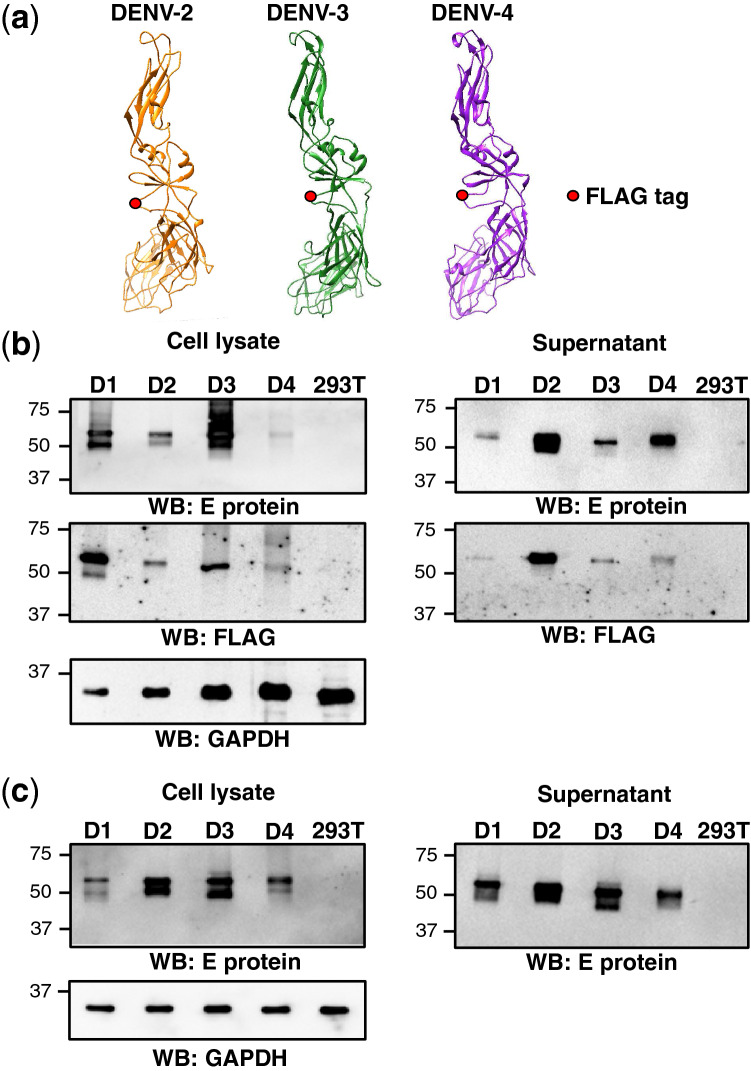
Expression of VLPs derived from all serotypes of DENV. (**a**) E protein structures of DENV-2, DENV-3 and DENV-4 were constructed with amino acid sequence of aa 1–395, aa 1–392 and aa 1–395, respectively. Positions of FLAG tag insertion at 273/274 in DENV-2 and -4 E protein and at 271/272 in DENV-3 E protein were shown with red circles. (**b**) Protein expression of FLAG inserted DENV-LPs derived from serotypes 1 (D1), 2 (D2), 3 (D3) and 4 (D4) VLP in cell lysates (left) and culture supernatants (right) were detected by western blotting. Non-transfected HEK293T samples (293 T) were added as negative controls. Amounts of cell lysates from D1 and D2 samples used were one-tenth compared to those from D3 and D4 as well as negative control. (**c**) Protein expression of DENV-LPs derived from serotypes 1, 2, 3 and 4 without FLAG tag in cell lysates (left) and culture supernatants (right) were detected by western blotting. Non-transfected HEK293T samples (293 T) were added as negative controls. Uncropped images of western blotting pictures in Fig. 2 are included in supplementary Fig. 2.

HEK293T cells transiently transfected with each prME construct were assessed for expression of processed E protein by western blotting. Among tested, the DENV-1 E protein with FLAG insertion at aa 273/274 (FL-273) was most efficiently expressed and secreted into the culture supernatants (Fig. 1c). As shown in the blotting with anti-glyceraldehyde-3-Phosphate Dehydrogenase (GAPDH) antibody as a loading control, it is noted that samples from FL-273 were diluted more than others before analyses (see legend of Fig. 1c). Further, the identified position, which is not required for multimerization of E protein and is localized on the surface of the virus particle, is predicted to be spatially conserved among all serotypes of DENV (Fig. 2a). Thus, to generate VLPs of DENV-2, DENV-3 and DENV-4 with FLAG tag, three prME expression plasmids carrying FLAG tag at the corresponding position 273/274 in E protein of DENV-2, DENV-4 and the position 271/272 in E protein of DENV-3, respectively, were constructed and their expression, together with DENV-1, was compared. Under the same transfection condition, the steady state level of E protein from DENV-1 and DENV-3 in cells was higher compared to that from DENV-2 and DENV-4 (Fig. 2b, left). In contract, the secreted protein level from DENV-2 was the highest, followed in order by DENV-4, DENV-3 and DENV-1 (Fig. 2b, right). Nonetheless, the results showed that a certain amount of FLAG-tagged E protein secreted was detectable in the cultures expressing either prME construct from DENV-1 to DENV-4.

As references, expression from original, non-tagged version of prME constructs was also tested by transient transfection as above (Fig. 2c). Although the E protein levels in cells and culture supernatants derived from DENV-2 and DENV-3 were to some extent higher than those from DENV-1 and DENV-4, their difference among DENV-1, DENV-2, DENV-3 and DENV-4 appeared limited compared in the case of FLAG tagged version. The results indicate that, while three-dimensional structures of E protein are largely similar among all four serotypes of DENV, an influence of the foreign epitope insertion on the protein expression and secretion is potentially observed dependent on the viral serotypes or isolates/clones.

### Purification and characterization of VLPs of DENV-1 to DENV-4 with FLAG insertion

**Figure 3:**
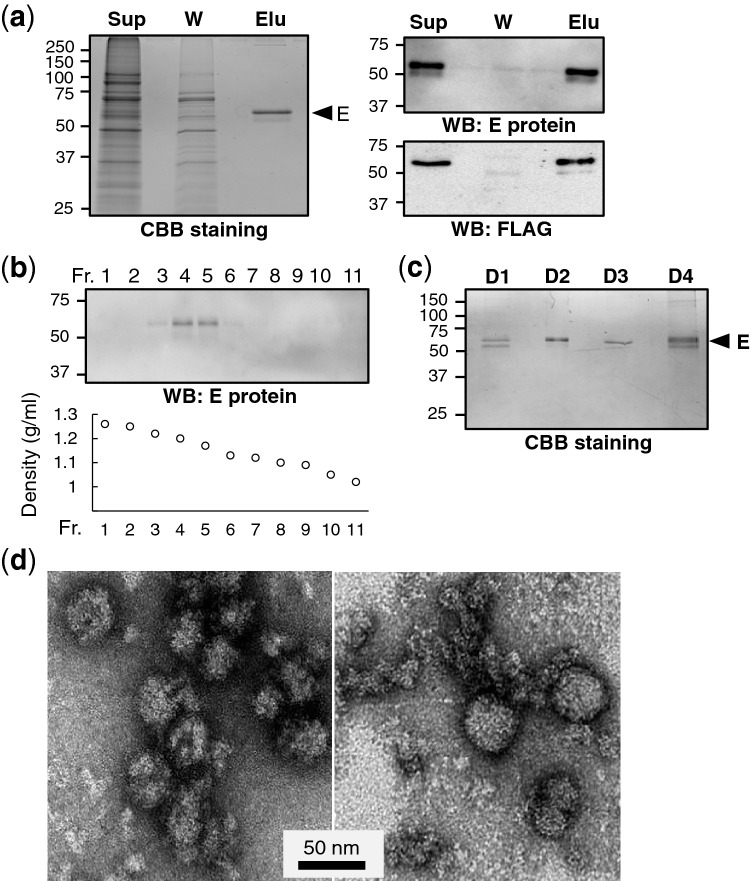
Purification and characterization of FLAG-inserted DENV-LPs. (**a**) FLAG-inserted DENV-2 VLP was purified by utilizing magnetic beads conjugated with the anti-FLAG antibody. Aliquots of crude culture supernatant from FL-273-expressing cells (Sup), washing buffer used after VLP-beads binding (W) and eluate from the VLP-bound beads (Elu) were separated by SDS-PAGE, followed by CBB staining (left) and western blotting with anti-E protein antibody (upper light) and anti-FLAG antibody (lower right). (**b**) The density of DENV-LP was determined by sucrose density gradient centrifugation. Aliquots of each fraction (Fr.1–11) were analyzed by SDS-PAGE and western blotting. (**c**) Purified FLAG-inserted VLPs derived from DENV-1, -2, -3 and -4 analyzed by SDS-PAGE and CBB staining. Uncropped images of western blotting and CBB staining pictures in Fig. 3 are included in supplementary Fig. 3. (**d**) DENV-2 VLP samples were stained with uranyl acetate solution and observed by TEM. The scale bar shows the length of 50 nm.

The DENV-2 VLP presenting FLAG tag in E protein was expressed in HEK293T cells and purified from culture supernatants by utilizing magnetic beads conjugated with an anti-FLAG (DYKDDDDK) antibody. The eluted fraction was subjected to SDS-PAGE and analyzed by Coomassie brilliant blue (CBB) staining, indicating a major nearly 55 kDa FLAG-DENV-2 E protein (Fig. 3a). The density of the purified DENV-LP was around 1.17–1.20 g/mL, which is similar to that of dengue virion reported^20,21^, as determined by sucrose density gradient centrifugation (Fig. 3b). VLPs with FLAG insertion derived from DENV-1, -3 and -4 were also purified in the same way (Fig. 3c). The yield of DENV-LPs after purification, as determined by comparing bands between serial dilutions of BSA and purified VLPs in CBB staining, were up to about 250, 1500, 750, and 500 mg/l (data not shown), respectively, for DENV-1, DENV-2, DENV-3 and DENV-4. Transmission electron microscopy of DENV-LPs displayed semi-smooth spherical particles with irregular sizes with diameters of 30–50 nm (Fig. 3d), which are similar to those previously reported^12,22^.

### Neutralizing activity of sera from mice immunized with tetravalent DENV-LPs

**Figure 4:**
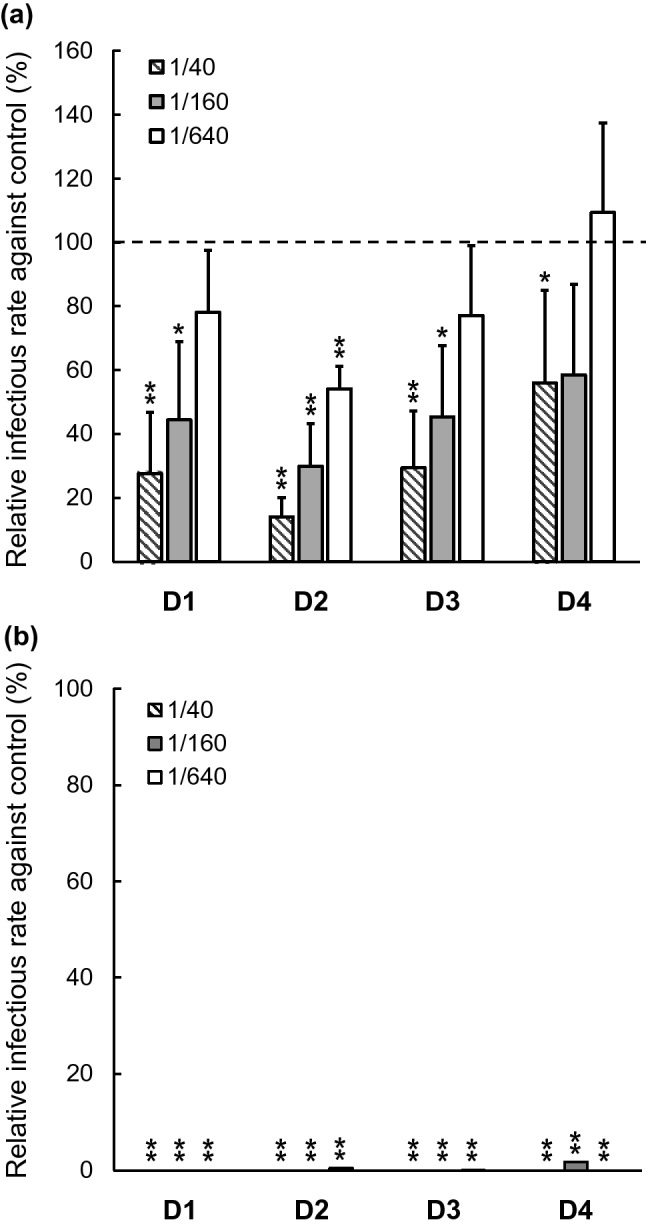
Evaluation of neutralization activity of mice sera immunized by tetravalent DENV-LPs. Infectious rates of DENV SRIPs in the presence of control sera were shown in the Supplementary Fig. 4. (**a**) Neutralizing activity of immunized sera against DENV-1 (D1), -2 (D2), -3 (D3) and -4 (D4) was individually evaluated by relative infectious rate using SRIPs assay based on the average of control, non-immunized mouse sera and was expressed as a relative infectious rate against control (100%) for each serum dilution. Dilution rates of test sera were 1:40 (diagonal lined bars), 1:160 (gray bars) and 1:640 (white bars). Statistical analyses for comparison between infectious rates of immunized (n = 4) and control sera (n = 5) were performed by Welch’s test (*p < 0.05, **p < 0.01). Error bars were drawn based on standard deviation. The dotted line indicates 100% of relative infectious rate based on the mean infectious rate of the control sera. (**b**) Neutralizing activity of Zika virus NS1 IgG-positive human serum used as a positive control was evaluated as described in (**a**). The positive control serum was tested in quadruplicate and statistically compared with negative control sera similarly (**p < 0.01).

To evaluate the neutralizing antibody response in mice induced by tetravalent DENV-LPs purified as described above, BALB/c mice were immunized with 40 µg of tetravalent DENV-LP mixture, which consists of 10 µg DENV-1 to DENV-4 VLPs individually, two times followed by the third injection with a half dose of DENV-1 to DENV-4 VLPs at 3-week intervals. Neutralizing activities of the immunized sera were assessed using four kinds of single-round infectious particles (SRIPs) possessing structural proteins derived from DENV-1 to DENV-4 individually. As shown in Fig. 4a, sera from immunized mice significantly reduced SRIP infection by all DENV serotypes. In particular, the 50% inhibitory dilution titer of the immunized sera against DENV-1, DENV-2 and DENV-3 were 1:160 or more dilution. A human serum positive with Zika virus IgG almost completely blocked the infection of any DENV SRIPs even at 1:640 dilution (Fig. 4b). These results thus demonstrated that our tetravalent dengue vaccine platform based on DENV-LPs with FLAG insertion elicited neutralizing antibody response against all serotypes concurrently.

## Discussion

Dengue is now the most frequent arboviral disease and represents an increasingly significant cause of morbidity in the world. One of the most difficulties to develop an effective dengue vaccine is the interaction among the four serotypes of DENV, leading to immunopathology of severe dengue. Although infection with one of the four serotypes potentially induces lasting protection against homotypic re-infection, secondary heterotypic infection is known to be associated with an increased risk of severe disease. Thus, vaccine development has been focusing on the generation of a tetravalent vaccine that enables to provide long-term protection against all four DENV serotypes.

In this study, we demonstrated an effective strategy to produce DENV VLPs containing the FLAG tag and to concentrate and purify them with one-step chromatography. The main reason for choosing FLAG tag was that we have found that VLP formation can be maintained when introduced into the E protein of JEV, a closely related virus to DENV^18^ and that techniques for highly specific one-step purification of fusion proteins by affinity pull-down using anti-FLAG agarose beads have been established. Characteristic features of purified VLP such as particle size, shape and density were basically comparable to flavivirus like particles reported previously^12,22,23^. The tetravalent dengue vaccine candidate, which consisted of purified VLPs derived from DENV-1 to DENV-4, was found to induce neutralizing responses against all serotypes of DENV. Further investigation should be focused on determining an optimal condition for induction of the protection activity against infection with DENV-1 to DENV-4.

Typical purification process of extracellularly produced flaviviral VLPs is made up of a variety of steps including centrifugation or depth filtration for cell debris removal and fractionation by density gradient centrifugation in combination with size-exclusion chromatography and ion exchange chromatography as lab-scale processes^11,12,24–26^. However, in general the ultracentrifugation step is not applicable to industrial-scale processes due to its limitation in scalability and variability. As affinity purification technology, immobilized metal ion adsorption chromatography with poly-histidine tag is a versatile and conventional method to purify the recombinant protein^25^ but it is known that problems such as low purity of the target proteins eluted from the column or unexpected elution during sample loading/washing frequently arise compared to the methods with specific antigen–antibody affinity purification. Compared to non-enveloped VLPs such as human papillomavirus and hepatitis E virus, enveloped VLPs appear to pose a challenge due to their lower stability. The FLAG tag pull-down technique might be advantageous for purification of enveloped VLPs, since VLPs presenting FLAG can be eluted in mild pH condition with the peptide solution.

In the first part of this study, we selected the aa residues 273/274 in the E protein as an optimal insertion site of FLAG tag for efficient production and secretion of DENV-1 derived VLP among three sites, aa 77/78, 223/224 and 273/274. Further, this VLP design carrying the FLAG tag was found to be applicable to DENV-2, DENV-3 and DENV-4 and allowed to develop a set-up for purification of DENV-LPs of all serotypes by affinity pull-down using anti-FLAG agarose beads. In designing where to insert the FLAG tag into the DENV-1 E protein, we referred to the results of a previous study on JEV, in which 11 sites for insertion of FLAG tag in the loop region of JEV E protein were tested for VLP production and three constructs were capable of production and secretion of JEV-LP^18^. Crystallographic studies have shown that the E protein consists of three structurally distinct domains: a central domain (domain I), a dimerization domain (domain II), and an immunoglobulin-like C terminal domain (domain III)^6^. The three sites used here were chosen from the viewpoints of predicted location; (1) in the exterior loop on the protruding domain of the E protein, (2) apart from the key determinant of neutralizing antibody (domain III) and (3) outside of dimerization interface of the protein. While located in domain II, formation of the E protein dimer is presumably not perturbed by a certain foreign epitope inserted at any of these sites, considering conformational states of the E protein. It may be likely that the FLAG peptide sequence insertion either at aa 77/78 or 223/224, rather than at aa 273/274, in the E protein resulted in impairing stability, dimer-dimer interaction and/or transportation during maturation of DENV-LP. A comprehensive structure–function analysis has shown that several residues relatively near aa 273 and 274 in the C terminal region of domain II such as aa 258 (methionine), 259 (histidine), and 265 (alanine) are critical for an efficient expression of DENV E protein and budding of the particles assembled^27^. In contrast to aa 273 and 274, it is supposed that these three critical residues are exposed on the underside of E protein and contact with the M protein residues. The efficiency of VLP production varied depending on DENV types/clones. The amino acid sequence homology between the four E proteins used in this study ranges from 40.5 (DENV-3 versus DENV-4) to 72.3% (DENV-1 versus DENV-3). Although the three-dimensional structures of the four E proteins are basically similar, there are some differences in the amino acid sequences, which may lead to differences in the stability of the E proteins and the efficiency of VLP assembly and/or secretion.

The on-going COVID-19 pandemic has indicated the importance of shortening timelines to develop vaccines effective for prophylaxis of emerging- and/or reemerging infectious diseases with global pandemic risk. Establishment of rapid one-step purification systems for VLP vaccine platforms, using tagging strategy, should be advantageous for vaccine development not only for dengue but for emerging pandemics. Our strategy would bear the potential to serve as a template for vaccine platform and be applied to development of VLP vaccines for other viruses, with minor adaptations.

## Materials and methods

### Plasmids

Expression plasmids for pre-membrane (prM) and envelope (E) protein of DENV-1 inserting FLAG tag sequence (DYKDDDDK) either at aa residues 77 and 78, 223 and 224, or 273 and 274, were generated as follows. DENV-1 prME expressing plasmid, pCAG_D1YG1_prME, was prepared by amplifying cDNA encoding prME of DENV-1 (D1/Hu/Saitama/NIID100/2014)^28^ and being cloned into pCAGGS. Then the linear FLAG tag inserted pCAG_D1YG1_prME was amplified by inverse PCR with the FLAG tag inserted PCR primer pair binding each position. The amplified liner FLAG tag containing plasmid DNA was self-ligated using In Fusion HD Cloning Kit (TaKaRa, Shiga, Japan), yielding pDENV1-FL77/78, -FL223/224, and -FL273/274.

prM/E expression plasmids for DENV-2, DENV-3 and DENV-4 with FLAG-tag insertion in E protein at aa 273 and 274 for pcD2ME^CO^ (DENV-2), pcD4ME^Th^ (DENV-4), and at aa 271 and 272 of pcD3ME^SG^ (DENV-3)^28^ which have two amino acids deletion before the insertion position, were similarly constructed and used as templates and cloning vectors, yielding pDENV2-FL273/274, pDENV4-FL273/274, and pDENV3-FL271/272, respectively.

### Homology modeling of DENV E protein structure.

The 3D structures of E protein derived from DENV-1 to DENV-4 were created from aa sequences used in this study by using SWISS-MODEL server (https://swissmodel.expasy.org)^29^. Crystal structures of E protein of DENV-1 (Protein Data Bank [PDB] ID: 4GT0), DENV-2 (PDB ID: 7BUB), DENV-3 (PDB ID: 1UZG) and DENV-4 (PDB ID: 3UAJ) were used as templates to build homology models. These structures were visualized and colored with UCSF Chimera 1.14 software (https://www.rbvi.ucsf.edu/chimera)^30^.

### Expression and purification of DENV-LPs.

Human embryonic kidney HEK293T cells, which were maintained in D-MEM with 10% of FBS, were transfected with prM/E expression plasmids using Lipofectamin LTX Reagent (Life technologies, Carlsbad, USA) or linear polyethylenimine, MW25000 (Thermo Fisher Scientific). To purify DENV-LPs, culture supernatants of the transfected cells were harvested 2–3 days post-transfection and then clarified by centrifugation. DYKDDDDK tag magnetic beads (Wako, Osaka, Japan) was added to the resulting supernatants and were rotated overnight at 4 ℃. Captured protein was released competitively using DYKDDDDK peptide (Wako, Osaka, Japan) with rotating for 1–2 h at 4 ℃. Purified VLP solutions were stored at − 80 ℃ before use.

### Protein electrophoresis and western blotting

Protein expression and purification was confirmed by SDS–polyacrylamide gel electrophoresis (PAGE) with 12.5% or 17% gel, followed by staining with Coomassie brilliant blue staining solution, EzStain Aqua (ATTO, Tokyo, Japan). For Western blotting, proteins after SDS-PAGE were transferred on PVDF membrane, followed by blocking with Block Ace (KAC Co., Ltd., Kyoto, Japan). Anti-flavi group antigen antibody (D1-4G2-4-15, Merck, Darmstadt, Germany), anti-DYKDDDDK tag antibody (Wako, Osaka, Japan) and anti-GAPDH antibody (Santa Cruz Biotechnology, Dallas, USA) were used as the primary antibodies. Membranes were then washed with Tris buffered saline containing 0.1% Tween 20 and treated with anti-mouse IgG HRP linked antibody (Cell Signaling Technology, Danvers, USA). Protein bands were stained with Amersham ECL Select (GE healthcare, Chicago, USA). Pictures of stained gels and membranes were obtained with Chemidoc touch (Bio-Rad, Hercules, USA).

### Transmission electron microscopy (TEM)

TEM analysis was performed as described previously^31^. In brief, VLP solutions were concentrated with Amicon ultra 0.5 centrifugal filter 10 k (Merck, Darmstadt, Germany) and exchange solvent to ultra pure water, followed by placing onto a formvar-coated copper grid and negatively stained with 2% uranyl acetate solution. The samples were observed by using a JEM-1400 plus electron microscope (JEOL Ltd., Tokyo, Japan) operating at an acceleration voltage of 100 kV. Digital images were captured by using a CCD camera.

### Immunization of mice

Female BALB/c mice (9 weeks of age) were purchased from SLC Japan and used for VLP immunization. Inoculums were prepared by equivalent volume of tetravalent VLP mixture containing purified VLPs derived from DENV-1 to DENV-4 and Titer Max Gold (TiterMax USA Inc., GA, USA) adjuvant were mixed. Similarly, saline was mixed with the adjuvant as a negative control. Mice (VLP group; n = 4, control group; n = 5) were immunized three times at 3-week intervals. The VLP mixture consisting of 10 µg of each VLP (DENV-1, -2, -3 and -4) was intraperitoneally inoculated at the first and second shots, and the mixture containing 5 µg of each VLP was inoculated at the third challenge. Serum samples were collected at 3 weeks after the third immunization. Mice used were euthanized by exsanguination under deep anesthesia using midazolam, medetomidine and butorphanol tartrate. All animal experiments were performed in accordance with the basic guidelines for animal experiments of the Ministry of Education, Culture, Sports, Science and Technology of Japan and in compliance with the Animal Research: Reporting of In Vivo Experiments (ARRIVE) guidelines. The study was approved by the Animal Experiment Committee of the Hamamatsu University School of Medicine (Approval Number: 2017079) and was carried out with the Regulation of Animal Experiments of the university.

### Neutralization test

Neutralizing antibody activity was evaluated by Single Round Infection Particles (SRIPs) assay^28^. Four kinds of SRIPs carrying M and E proteins derived from DENV-1, -2, -3, and -4 individually were prepared as described^28^. HEK293T cells were co-transfected with three plasmids; a DENV-1-derived subgenomic replicon plasmid containing nanoluciferase gene, a DENV-1 capsid-expression plasmid, and a plasmid exprerssing prME either derived from DENV-1, -2, -3 or -4. The culture media were harvested after 3 days of transfection and used as SRIPs carrying each type of DENV envelope. Serially-diluted immunized sera were mixed with each SRIP (approximately 100 infectious unit/well) at a 1:1 ratio, followed by adding to monolayers of African green monkey kidney Vero cells. After 3 days of incubation, luciferase activity in cells was determined using the Nano-Glo Luciferase Assay System (Promega, WI, USA). The neutralization titer of immunized serum was evaluated by relative inhibition rate of SRIPs infection compared to the mean inhibition rate of control serum in each dilution. Zika virus NS1 IgG-positive human serum samples were purchased from TRINA BIOREACTIVES AG (Switzerland) and used as a positive control for neutralization.

### Statistical analysis

Statistical comparison between two study groups in the neutralization test was analyzed by Welch’s test using R software version 3.0.0^32^. P values less than 0.05 were determined as significantly difference.

## Supplementary Information

Supplementary Figures.
