# Supplementary material for: Virus-like particles with FLAG-tagged envelope protein as a tetravalent dengue vaccine candidate
Source: Sci Rep. 2021 Sep 2;11:17542. doi: 10.1038/s41598-021-97038-4 (PMC8413300; doi:10.1038/s41598-021-97038-4)

Virus-like particles with FLAG-tagged envelope protein as a tetravalent dengue vaccine candidate

Toshifumi Imagawa[1], Masahiko Ito[1], Mami Matsuda[2], Kenji Nakashima[1], Yuhei Tokunaga[3], Isao Ohta[3], Tian-Cheng Li[2], Ryosuke Suzuki[2], Tetsuro Suzuki[1]*

1 Department of Virology and Parasitology, Hamamatsu University School of Medicine, Hamamatsu 431-3192, Japan
2 Department of Virology II, National Institute for Infectious Disease, Musashi-murayama, Tokyo 208-0011, Japan
3 Advanced Research Facilities and Services, Preeminent Medical Photonics Education & Research Center, Hamamatsu University School of Medicine, Hamamatsu 431-3192, Japan

This file contains supplementary figure 1 to 4.

Supplementary figure 1

Uncropped pictures of western blotting in Figure 1

Figure 1(c)

Cell lysate

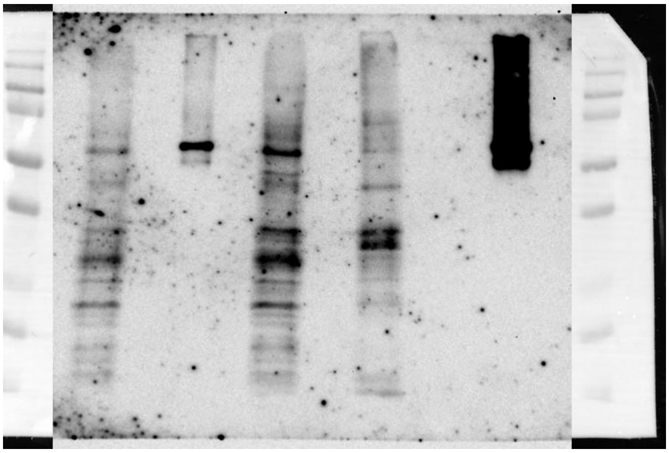

WB:FLAG

Supernatant

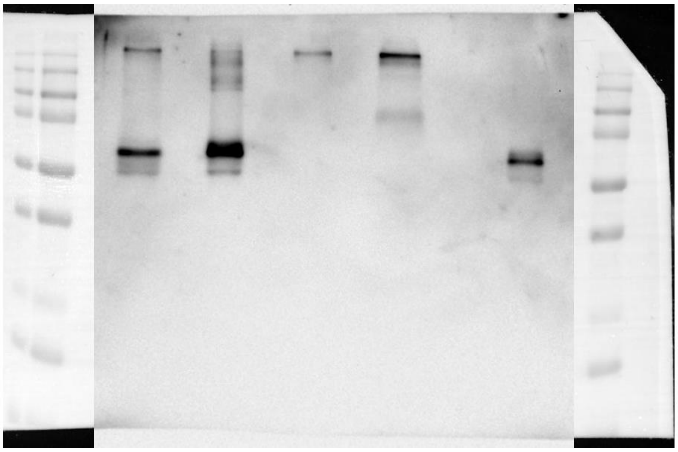

WB:E protein

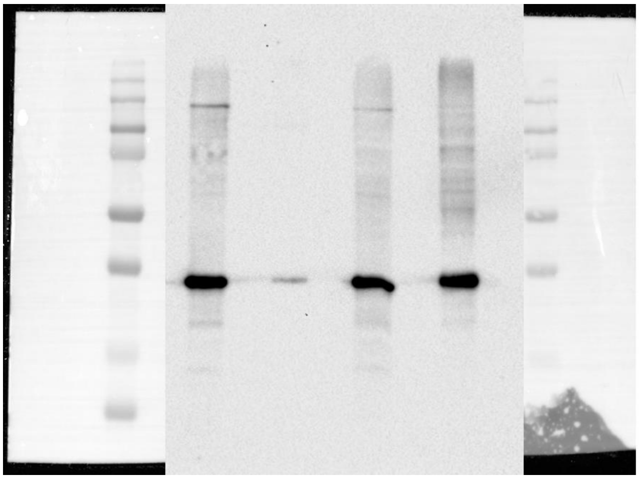

WB: GAPDH

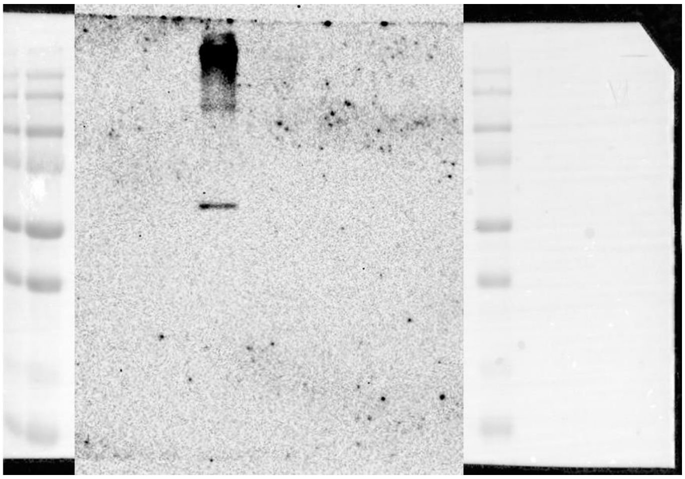

WB:FLAG

Supplementary figure 2

Uncropped pictures of western blotting in Figure 2

Figure 2(b)

Cell lysate

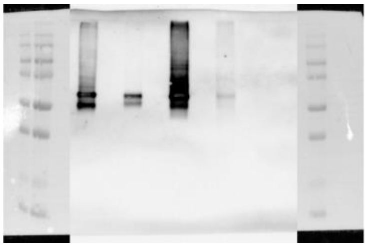

WB:E protein

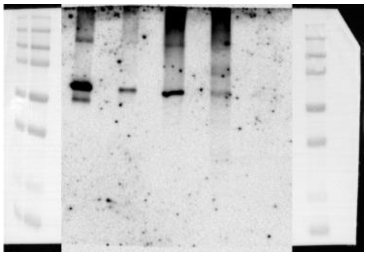

WB:FLAG

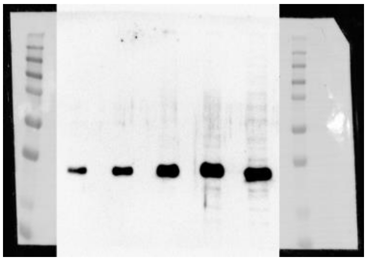

WB: GAPDH

Supernatant

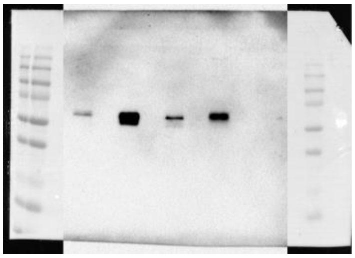

WB:E protein

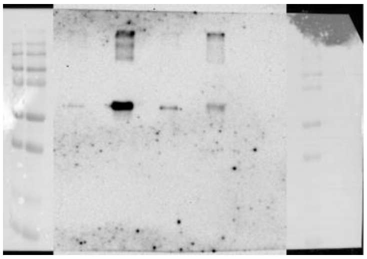

WB:FLAG

Figure 2(c)

Cell lysate

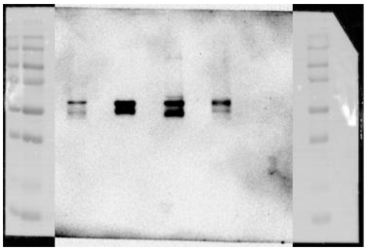

WB:E protein

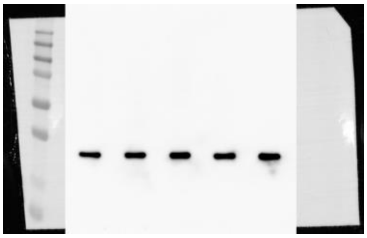

WB: GAPDH

Supernatant

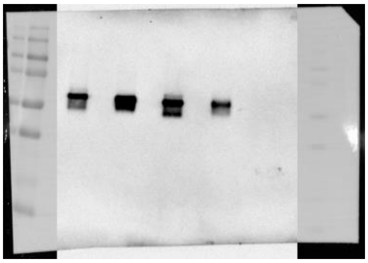

WB:E protein

Supplementary figure 3

Uncropped pictures of western blotting and CBB staining in Figure 3

Figure 3(a)

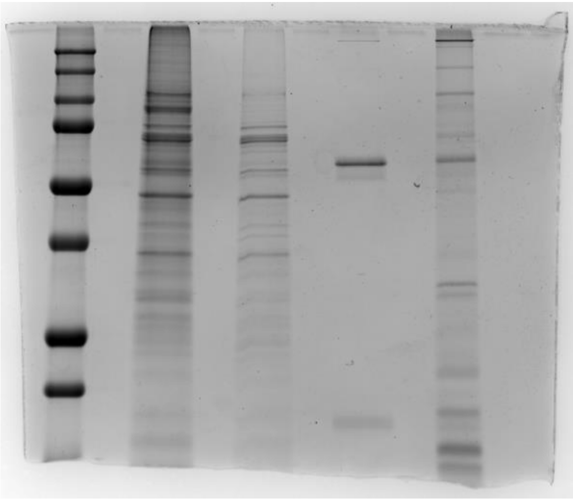

CBB staining

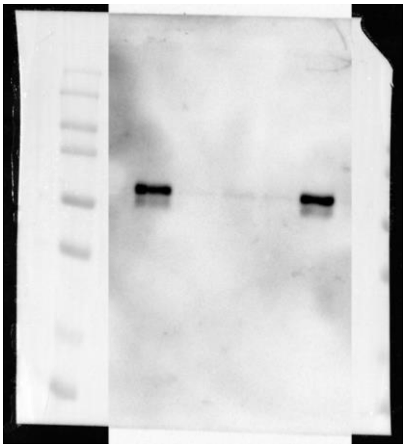

WB: E protein

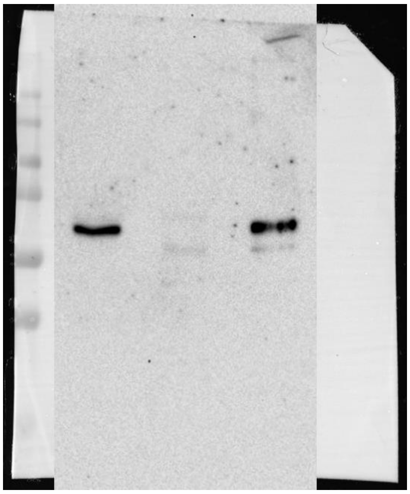

WB: FLAG

Figure 3(b)

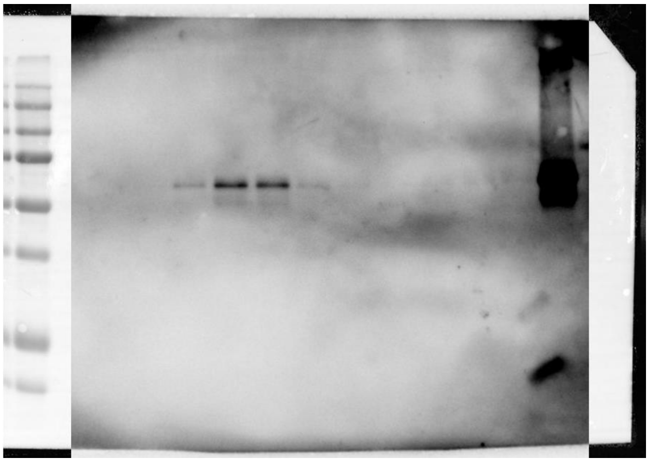

WB: E protein

Figure 3(c)

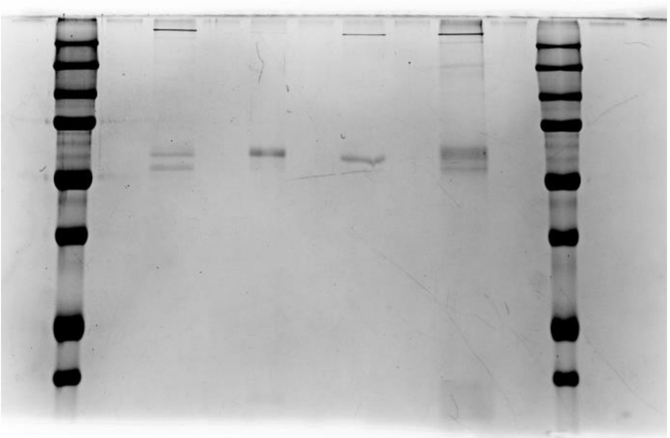

CBB staining

Supplementary figure 4

Infectious rates of DENV SRIPs in the presence of diluted sera from each mouse (C1-C5) in non-immunized, negative control group were shown.

DENV-1

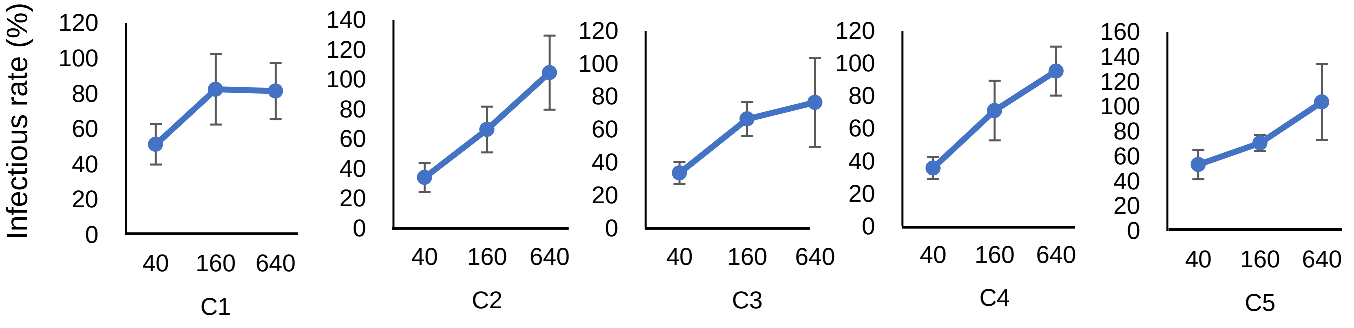

DENV-2

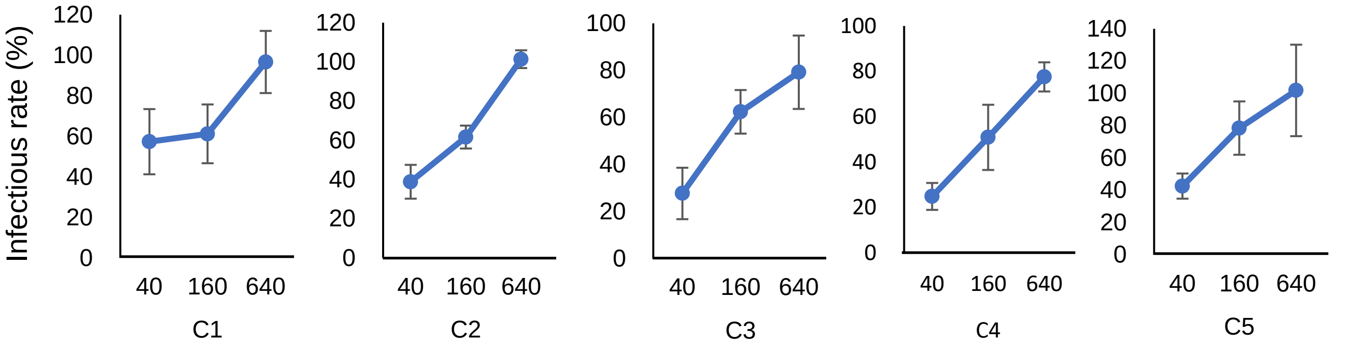

DENV-3

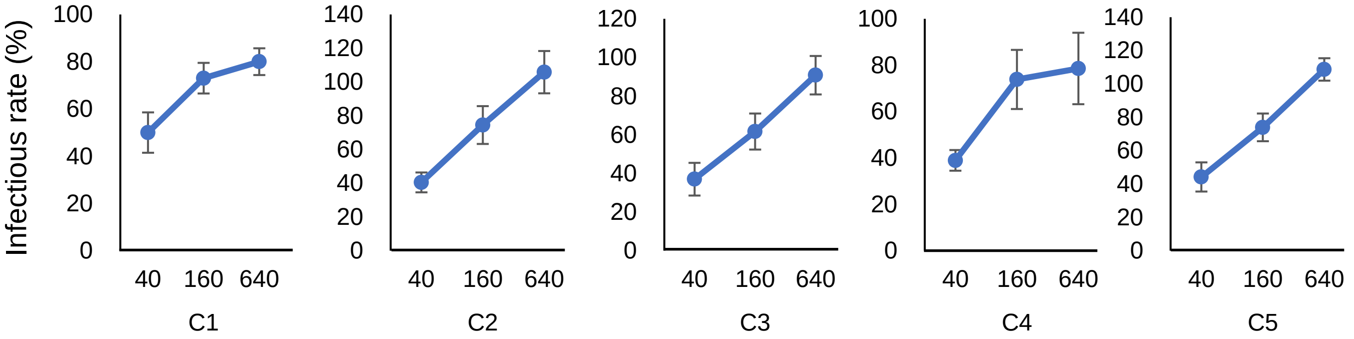

DENV-4

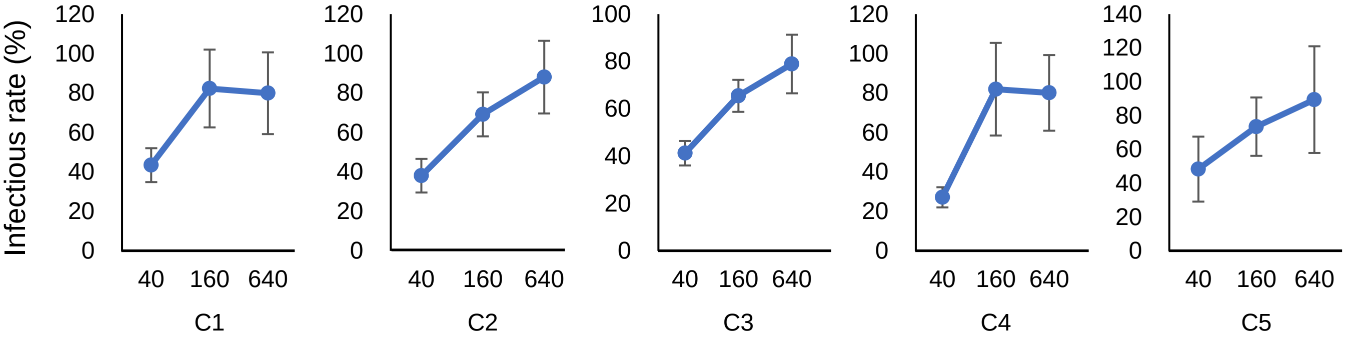

Supplement: Supplementary file 1 — Supplementary Figures. [file 41598_2021_97038_MOESM1_ESM.pdf]
